# Temporomandibular Joint Osteoarthritis: Pathogenic Mechanisms Involving the Cartilage and Subchondral Bone, and Potential Therapeutic Strategies for Joint Regeneration

**DOI:** 10.3390/ijms24010171

**Published:** 2022-12-22

**Authors:** Anca Cardoneanu, Luana Andreea Macovei, Alexandra Maria Burlui, Ioana Ruxandra Mihai, Ioana Bratoiu, Ioana Irina Rezus, Patricia Richter, Bogdan-Ionel Tamba, Elena Rezus

**Affiliations:** 1 Department of Rheumatology, “Grigore T. Popa” University of Medicine and Pharmacy, 700115 Iasi, Romania; 2 Clinical Rehabilitation Hospital, 700661 Iasi, Romania; 3 Faculty of Medicine, “Grigore T. Popa” University of Medicine and Pharmacy, 700115 Iasi, Romania; 4 Advanced Research and Development Center for Experimental Medicine (CEMEX), “Grigore T. Popa” University of Medicine and Pharmacy, 16 Universitatii Street, 700115 Iasi, Romania; 5 Department of Pharmacology, Clinical Pharmacology and Algesiology, “Grigore T. Popa” University of Medicine and Pharmacy, 16 Universitatii Street, 700115 Iasi, Romania

**Keywords:** osteoarthritis, temporomandibular joint, bone remodeling, chondrocyte death, regenerative therapy

## Abstract

The temporomandibular joint (TMJ) is a specialized synovial joint that is crucial for the movement and function of the jaw. TMJ osteoarthritis (TMJ OA) is the result of disc dislocation, trauma, functional overburden, and developmental anomalies. TMJ OA affects all joint structures, including the articular cartilage, synovium, subchondral bone, capsule, ligaments, periarticular muscles, and sensory nerves that innervate the tissues. The present review aimed to illustrate the main pathomechanisms involving cartilage and bone changes in TMJ OA and some therapeutic options that have shown potential restorative properties regarding these joint structures in vivo. Chondrocyte loss, extracellular matrix (ECM) degradation, and subchondral bone remodeling are important factors in TMJ OA. The subchondral bone actively participates in TMJ OA through an abnormal bone remodeling initially characterized by a loss of bone mass, followed by reparative mechanisms that lead to stiffness and thickening of the condylar osteochondral interface. In recent years, such therapies as intraarticular platelet-rich plasma (PRP), hyaluronic acid (HA), and mesenchymal stem cell-based treatment (MSCs) have shown promising results with respect to the regeneration of joint structures or the protection against further damage in TMJ OA. Nevertheless, PRP and MSCs are more frequently associated with cartilage and/or bone repair than HA. According to recent findings, the latter could enhance the restorative potential of other therapies (PRP, MSCs) when used in combination, rather than repair TMJ structures by itself. TMJ OA is a complex disease in which degenerative changes in the cartilage and bone develop through intricate mechanisms. The regenerative potential of such therapies as PRP, MSCs, and HA regarding the cartilage and subchondral bone (alone or in various combinations) in TMJ OA remains a matter of further research, with studies sometimes obtaining discrepant results.

## 1. Introduction

The temporomandibular joint (TMJ) is a synovial joint that allows mandibular motion relative to the cranial base and distributes normal function (chewing and speaking) and parafunction stresses (clenching and bruxism). The ability of condylar cartilage to rebuild in response to changes in condylar repositioning, articular function, and particular mechanical loading is the most remarkable biological characteristic that distinguishes it from other types of cartilage [1,2,3,4,5,6,7,8].

Unlike hyaline articular cartilage, which solely contains type II collagen, TMJ mandibular condyle fibrocartilage contains both type I and type II collagen [9]. Because of this, TMJ demonstrated enhanced healing capability and interstitial cartilage development. Another distinguishing characteristic of TMJ is that, in contrast to the articular cartilage in other joints, which is a primary cartilage, the cartilage of the mandibular condyle is a secondary cartilage [10,11,12]. The most frequent type of arthritis affecting TMJ is temporomandibular joint osteoarthritis (TMJ OA), which has a significant clinical prevalence and adverse effects on the TMJ [7,13]. TMJ OA is a chronic degenerative disease that affects both the cartilage and the subchondral bone [14]. TMJ OA is primarily characterized by chondrocyte death, extracellular matrix (ECM) degradation, and subchondral bone remodeling [13]. 

In recent years, several therapeutic strategies have been tested beyond their ability to improve OA-related symptoms, with research focusing on their regenerative potential. In this respect, platelet-rich plasma (PRP), hyaluronic acid (HA), and mesenchymal stem cells (MSCs) (either alone, or in various combinations) have shown some promising results with regard to the restoration of the normal cartilage and bone aspect in OA (or a delayed progression of disease-related changes), as shown by objective assessment methods of their effects (such as imaging techniques or histological examination) [1,3]. Nevertheless, studies report sometimes discrepant findings.

The present review aimed to illustrate the main pathomechanisms involving cartilage and bone changes in TMJ OA and some therapeutic options that have shown potential restorative properties regarding these joint structures in vivo. We conducted a search of the literature regarding the pathogenesis of cartilage and bone degeneration in TMJ OA. In order to present the role of PRP, HA, and MSCs in the treatment of TMJ OA, we researched original articles published between 2019–present using the keywords “temporomandibular” and “osteoarthritis” and “platelet-rich plasma”/”hyaluronic acid”/”mesenchymal stem cells”. Of the resulting searches, we read and selected the articles that involved either experimental animals or human subjects with degenerative TMJ involvement and used objective measures to investigate cartilage and bone changes (imaging techniques, histological assessment). 

## 2. Cartilage Involvement in Temporomandibular Joint Osteoarthritis

Mechanical factors that cause excessive or unbalanced joint loading influence the onset and progression of TMJ OA. Mechanical factors include injuries (which cause a change in the mechanical properties of the articular disc, cartilage degradation, and the production of inflammatory and pain mediators), parafunctions (which determine articular disc dislocation and degenerative changes within the condyle and articular eminence), increased friction within the TMJ, unstable occlusion, and functional overload [15]. In addition to the local causes, systemic risk factors for TMJ OA include aging, female sex, metabolic and autoimmune illnesses, dietary inadequacies, hormonal variables, and a predisposed genetic profile [16].

Degenerative changes result from a disruption in the remodeling of the TMJ. Remodeling is the essential biological response to TMJ loading. It maintains the balance of the joint, function, and occlusion. Excessive or prolonged TMJ overload and a reduction in TMJ adaptability may result in incorrect remodeling [17].

The degradation of collagen and proteoglycans in cartilage leads to fibrillation, erosion, and cracking in the superficial cartilage layer [18]. This process spreads to a deeper layer of cartilage and enlarges to generate erosions over time. The articular surface of the TMJ exhibits exceptional adaptability. The hyaline cartilage of the body’s weight-bearing joints is more robust to compressive loading, whereas the fibrocartilage of the TMJ is more resistant to shear force [19].

Early cartilage deterioration may be caused by metabolic or mechanical causes. It is a sequence of biomechanical changes in the joint’s hard and soft tissues, which in turn triggers the immunological response. Immune cells initiate an inflammatory response by releasing cytokines and chemokines, among other inflammatory mediators. The process is accompanied by the activation of the complement system and the production of cartilage-degrading molecules, including matrix metalloproteinases (MMPs) and prostaglandin E (PGE), further deteriorating the articular cartilage. This leads to the eventual degeneration and abrasion of joint cartilage and the remodeling of the subchondral bone through the onset of a local inflammatory response [17,20].

The defining characteristics of TMJ OA are both radiological and clinical [21]. The clinical manifestations include tenderness in the joint region, pain during mouth opening and lateral excursion, and grating or crepitus. Cortical bone degradation, flattening of joint compartments, and productive bone alterations such as sclerosis and osteophytes are radiographic manifestations of the disease [22].

As cartilage’s resident cells, chondrocytes are the essential mediators in maintaining the homeostasis of the cartilage matrix. Compromised chondrocyte activity and survival would disrupt cartilage homeostasis and hasten the progression of OA [9].

The quality of condylar bone is strongly related to the development of TMJ osteoarthritis. The gradual cartilage degeneration is caused by improper regulation of chondrocytes and an imbalance between tissue destruction and production [14]. Chondrocytes regulate the equilibrium between ECM synthesis and breakdown. However, this balance can be disrupted if catabolic and synthesis activity is not connected [23]. In addition, the number of apoptotic chondrocytes increases dramatically in OA, which is directly associated with the endoplasmic reticulum and death receptor pathways [24].

Hypertrophic chondrocytes induce the breakdown of the ECM and calcification of cartilage. This denatured cartilage adapts less mechanically to damaging stimuli, such as trauma [25]. Additionally, angiogenesis has been implicated in TMJ OA [14,26].

### 2.1. Chondrocyte Apoptosis 

Multiple studies have shown that an increase in chondrocyte turnover initiates the deterioration of condylar cartilage [27]. Apoptosis, autophagy, and necroptosis are important in chondrocyte death. Apoptosis and necroptosis promote articular cartilage degeneration [14]. Chondrocyte apoptosis creates space for neovascularization, and it is believed that the apoptotic bodies created by this process constitute the origin of cartilage mineralization [25].

Calcium is essential for chondrocyte death, and mechanical strain can raise calcium concentration in chondrocytes [28]. Endoplasmic reticulum stress (ERS) induced by calcium influx can cause death in chondrocytes; this phenomenon is called ERS-mediated apoptosis [29]. A high concentration of intracellular calcium can activate inducible nitric oxide synthase (iNOS). By releasing cytochrome C (Cyt C) and caspase-9, nitric oxide (NO) generated by iNOS limits mitochondrial respiration and leads to chondrocyte death [30].

In addition, tumor necrosis factor (TNF) and fibroblast growth factor receptor 1 (FGFR1) can promote chondrocyte apoptosis by working on the death receptor pathway [31]. Necroptosis is an important phenomenon in OA and is caused by oxidative stress. The breakdown of the cartilage is accelerated by necroptosis, which is mediated by TNF and receptor-interacting proteins 1 and 3 (RIP1/RIP3). According to studies, inhibiting apoptosis promotes the necroptosis pathway [32].

Chondrocytes recycle or reuse large macromolecules through a process known as autophagy, which is believed to be a self-protective mechanism. In most cases, aberrant mortality of chondrocytes not only results in a decrease in the total number of chondrocytes but also initiates the degeneration of cartilage and the breakdown of subchondral bone [33].

Autophagy is a crucial survival strategy for chondrocytes in OA [33]. The primary step of autophagy is the production of autophagosomes, which sequester organelles or macromolecules that have been eliminated. Eventually, autophagosomes merge with lysosomes to generate autolysosomes, which destroy the stored materials and release tiny molecules that can be reused. In the early stage of TMJ OA, the autophagy markers beclin 1 and light chain 3 beta (LC3B) rise, but they decrease considerably in the late phase [24]. Early autophagy increases and protects chondrocytes against environmental alterations. Cartilage destruction is connected with the suppression of autophagy and cell death [24,34].

Furthermore, the endoplasmic reticulum-associated proteins ERN1, mTORC1, and EIF2AK3 trigger apoptosis and limit autophagy. The ERN1–MTORC1–EIF2AK3 signaling axis is the name given to this pathway [24]. As a result, controlling autophagy may be a valuable method for treating TMJ OA.

### 2.2. ECM Degeneration

The cartilage ECM is mainly made up of collagen fibers and big proteoglycans. It not only serves as a protective structure for cartilage against elastic and shear pressures, but it also governs chondrocyte behavior via matrix–cell interactions [35].

MMPs and a disintegrin and metalloproteinase production with thrombospondin motifs (ADAMTS) initiate ECM breakdown in TMJ OA. Through the bone morphogenetic protein (BMP) pathway, the degradation of type II collagen (Col2A1) promotes the hypertrophy of chondrocytes, hence accelerating the course of TMJ OA [36]. In addition, cartilage mineralization has been demonstrated to contribute to the development of TMJ OA [25].

Activation of 2A-adrenergic receptor signals through the extracellular regulated protein kinases 1 and 2 (ERK1/2) and protein kinase A (PKA) pathways increases the synthesis of matrix degradation-associated enzymes, such as MMP-3 and MMP-13. Osteopontin, an inflammatory agent, stimulates the production of MMPs through the NF-κB signaling pathway [37]. 

The HTRA1–DDR2–MMP-13 axis is essential for ECM breakdown. This process starts with the overexpression of high-temperature requirement A1 (HTRA1) and the breakdown of pericellular matrix components, including type VI collagen. Col2A1 can activate the transmembrane protein Discoidin Domain Receptor Tyrosine Kinase 2 (DDR2) in the absence of a pericellular matrix. DDR2 ultimately triggers MMP-13 and hastens TMJ OA [38].

### 2.3. Molecular Signaling in the Development of TMJ OA

#### 2.3.1. Wnt/β-Catenin Signaling Pathway

The Wnt/β-catenin signaling pathway has been researched for decades in stem cell self-renewal, cell proliferation and differentiation throughout embryonic development, and adult tissue homeostasis. It is a conserved cellular communication system that influences the onset of OA and other forms of arthritis [9,39].

Intracellular β-catenin is stabilized and translocated to the nucleus thanks to a complex formed by Frizzled and low-density lipoprotein receptor-related proteins 5 or 6 (LRP5/6). Signaling through the Wnt/β-catenin pathway begins at the cell surface when the Wnt glycoprotein binds to specific receptors. After attaching to nuclear transcription factors, β-catenin protein allows for expressing Wnt-targeted proteins [9].

Progressive TMJ abnormalities, joint space narrowing, and OA-like TMJ defects have been documented in β-catenin conditional activation mice, β-catenin(ex3) Agc1ER. MMP-13, ADAMTS-4, and ADAMTS-5, which promote cartilage breakdown, were highly raised, whereas Col-X, a chondrocyte hypertrophy-related protein, was similarly upregulated in β-catenin(ex3) Agc1ER mice [9].

Furthermore, lower cell proliferation and higher cell death were found in these mice’s condylar cartilage. This evidence suggests that β-catenin plays a crucial role in TMJ pathophysiology and that Wnt/β-catenin signaling is a possible therapeutic target for treating TMJ OA [40].

#### 2.3.2. TGF-β and BMP Signaling 

Transforming growth factor β (TGF-β)/BMP signaling has been widely investigated concerning bone formation. It performs a variety of functions throughout life. The TGF-β superfamily consists of more than forty members, including TGF-βs, BMPs, and activin. They are embedded in the bone matrix and govern bone remodeling or influence the production of bone and cartilage [9,41].

The TGF-β signal pathway initiates intracellular signaling following the creation and activation of a heteromeric complex of types II and I serine/threonine kinase receptors, followed by the phosphorylation of particular Smad proteins, R-Smads. The phosphorylated R-Smads can heterodimerize with co-Smad, Smad4, ultimately translocating to the nucleus and activating the transcription of target genes [42].

In the TGF-β/Smad3 signaling pathway, sphingosine 1-phosphate (S1P), a bioactive lipid, is produced to function as an intracellular mediator or extracellular ligand for different receptors, resulting in inflammation, cell migration, and angiogenesis. The interaction between TGF-β/Smad3 and S1P/S1P3 and Smad3/S1P3 signaling in chondrocytes may play a role in the development of TMJ OA [43].

Additionally, it has been documented that overexpressing TGF-β1 causes aberrant subchondral bone remodeling that causes mice to develop TMJ OA and degrade mandibular condylar cartilage [9].

#### 2.3.3. Indian Hedgehog Signaling 

Indian hedgehog (Ihh), a signaling molecule, is essential for controlling the formation of the skeleton. During endochondral ossification, it is mainly expressed in pre-hypertrophic and hypertrophic chondrocytes. The production of parathyroid hormone-related protein (PTHrP) in periarticular tissue is one of the processes it controls during cartilage growth [44].

In TMJ osteoarthritic cartilage stimulated by unilateral anterior cross-bite (UAC), enhanced Ihh signaling promotes the terminal differentiation of deep zone chondrocytes. In contrast, the OA-like lesions and UAC-promoted chondrocyte terminal differentiation were rescued by the deletion of Smo in mice [9]. Another research found that Ihh facilitated OA development by controlling genes involved in cartilage deterioration and that Ihh inhibition mitigated the disease. Activation of Ihh, Smo, and Gli1 was detected in adjuvant-induced TMJ OA in mice [45], suggesting that Ihh signaling may exacerbate TMJ OA by encouraging chondrocyte hypertrophy.

#### 2.3.4. FGF Signaling 

Articular cartilage and the control of skeletal development are two of the essential functions of the FGF signaling system. There are 22 ligands in the FGF family, all of which bind to one of four different FGFRs to perform various tasks [46]. The binding of FGFs to the FGFRs’ extracellular domain is the usual starting point for FGF/FGFRs signaling. After the phosphorylation of the cytoplasmic tail of FGFRs and the recruitment of the corresponding target proteins, several different signaling processes are triggered.

Several signaling pathways are involved in the downstream signaling activities of FGF [47]. These include the phosphoinositide 3-kinase(PI3K)/Akt pathway, the phospholipase C (PLC) pathway, the mitogen-activated protein kinase (MAPK) pathway, and the signal transducers and activators of transcription (STAT) 1/p21 pathway. In addition, the FGF signaling pathway is connected with the development of OA via MEK/ERK, a critical downstream signaling molecule of FGFR1 [9].

FGF signaling may be increased in TMJ OA, as evidenced by the finding that ablation of FGRF1 in TMJ chondrocytes reduced TMJ OA development in particular OA models [48].

#### 2.3.5. NF-κB Signaling 

RelA, RelB, c-Rel, NF-κB1, and NF-κB2 are the five proteins that make up the NF-κB family. They interact with NF-κB inhibitors, form active complexes, move into the nucleus, bind to DNA, and control the expression of NF-κB-target genes [9]. The immune system, the inflammatory process, stress responses, cell proliferation, and cell death are all thought to be partially mediated by NF-κB.

The activation of the IKK-α/IKK-β/IKK-γ-NEMO complex is mediated by the TNF receptor (TNF-R), Toll-like receptor (TLR), or T-cell receptor (TCR) in the classical NF-κB signaling pathway. Inactive NF-κB dimers are linked to inhibitory NF-κB (I-κB) proteins in the cytoplasm. When mechanical and chemical cues stimulate cells, I-κBs are phosphorylated by I-κB kinases and degraded by the ubiquitin–proteasome system, allowing NF-κB heterodimers to translocate into the nucleus and promote the expression of target genes [9,49].

#### 2.3.6. Notch Signaling 

Necessary for cell differentiation and death, the Notch receptor is a single-pass transmembrane receptor at the cell surface. The highly conserved Notch signaling system consists of many components, including Notch ligands, Notch receptors, transcriptional effectors, and target genes [50]. Notch ligands bind to Notch receptors to commence the process, after which Notch receptors are cleaved, the intracellular domain of Notch receptors translocates to the nucleus, and target genes are activated [51].

The Notch signaling system controls the molecules involved in cartilage production and breakdown and hence plays a dual function in cartilage maintenance [52]. It is well known that Notch signaling is critical in the angiogenesis of condylar cartilage and disc, which is required to form TMJ OA [53]. Recent research has shown that changing Notch signaling may cause TMJ OA.

### 2.4. Angiogenesis in TMJ Osteoarthritis 

Angiogenesis has been shown to enhance the development of OA [54]. Wang and colleagues discovered that mice with TMJ OA-like alterations increased the number of newly created blood vessels at the osteochondral junction [53]. In TMJ OA, these new blood vessels can carry inflammatory mediators and prolong inflammation [55]. When new blood arteries enter the cartilage, they stimulate chondrocyte enlargement and mineral deposition in the matrix. Through endochondral osteogenesis, osteophytes can integrate with newly created vessels on the joint’s surface to enhance complex tissue creation [14].

Vascular endothelial growth factor (VEGF) is important for angiogenesis and a key modulator of TMJ OA. Several transcription factors, including hypoxia-inducible factor-1 (HIF-1), promote VEGF expression [14,56]. The protein dickkopf-related protein-1 (DKK-1) was discovered in high concentrations in the synovial fluid of TMJ OA patients. It has been proposed that DKK-1 and high-mobility group box 1 (HMGB1) guide HIF-1 nuclear localization, enhancing VEGF production [56,57]. Interleukin-6 (IL-6) and IL-1 elevate VEGF levels via stimulating VEGF transcription in the nucleus. ERK1/2 stimulates the estrogen-related receptor γ (ERR γ). IL-1, on the other hand, directly stimulates NF-κB [26,58].

VEGF is generated by chondrocytes in articular cartilage and modulates autocrine levels of MMP-13 and tissue inhibitor of metalloproteinase-1 (TIMP-1). Reduced TIMP concentration and increased MMP expression disrupt the circulation of ECM components, collagen, and proteoglycans, which is shown by an increased breakdown. VEGF may increase articular cartilage deterioration by activating osteoclasts and allowing blood vessels to penetrate the cartilage [15,17].

Due to the breakdown of hyaluronic acid (HA) and the increased activity of free radicals, joint hydration is also reduced. When the pressure within a joint begins to surpass the capillary pressure, transient hypoxia and joint degeneration ensue. Reoxygenation is detected when the stress on the joint is reduced, and joint degeneration is halted.

During hypoxia and reperfusion cycles, free radicals are released. Free radicals hinder production and accelerate HA breakdown, lowering synovial fluid viscosity and increasing friction between joint surfaces [59]. Increased friction during TMJ movement causes permanent joint structure damage, internal articular disc derangement, and degenerative alterations [15,60].

## 3. Subchondral Bone Changes in Temporomandibular Joint Osteoarthritis

The main pathogenic mechanism highlighted at the level of the subchondral bone in TMJ OA is abnormal bone remodeling. This is due to complex mechanisms that include mechanical loading, inflammation, and degradation of the articular cartilage [9,13,61,62]. The onset of degenerative changes is characterized by a loss of bone mass which acts as a trigger for the development of TMJ OA and which participates in the degradation of articular cartilage [63]. Then follows slow bone repair mechanisms that cause an increase in bone density at the subchondral level, which leads to increased thickening and stiffness of the condylar osteochondral interface [64,65]. Erosions, osteophytes, the appearance of cyst-like lesions, and subchondral sclerosis represent the main radiographic changes evident in TMJ OA [20,61,66,67]. Clinically, patients present a decrease in mandibular mobility, pain during mastication and with the opening of the oral cavity, as well as joint sounds (cracking) during TMJ mobilization [68].

### 3.1. Bone Cells in Temporomandibular Osteoarthritis

Bone remodeling in TMJ OA is associated with a decrease in the number and activity of osteoblasts, as well as an increase in osteoclast activity [63,64]. At the level of osteoblasts, bone-forming cells, a multitude of metabolic mechanisms with increased activity have been observed that favor angiogenesis, osteoclastogenesis, and subchondral bone sclerosis [69,70]. Thus, an increase in alkaline phosphatase (ALP) activity and an elevated expression of receptor activator of nuclear factor kappa-Β ligand (RANKL), transforming TGF-β1, VEGF, and insulin-like growth factor-1 (IGF-1) were highlighted in TMJ OA [71,72,73,74]. Moreover, osteoblasts participate directly in the formation of subchondral sclerosis characterized by an increase in bone density and volume, but associated with deficient mineralization [75,76]. This is due to the particular phenotype of osteoblasts that secrete abnormal type I collagen and that show increased expression of TGF-β which inhibits mineralization by stimulating the secretion of DKK2 [77,78]. 

Osteoclasts, the cells involved in bone resorption, present an increased number and intense activity in TMJ OA, the main activating mechanism of osteoclastogenesis being the binding of RANK with its ligand (RANKL) [79,80]. The most important degradative enzyme produced by osteoclasts that favors bone resorption is cathepsin K (CTSK), animal studies highlight its important role in the development of OA [81]. In addition, the migration and differentiation of osteoclast precursors are achieved through the WNT5A/receptor tyrosine kinase-like orphan receptor 2 (Ror2) pathway [82]. The activation of adrenergic receptors (β2 and α2A) via neurotransmitters determines, also through the RANKL pathway, the maturation of osteoclasts, and bone destruction [83]. Last but not least, osteoclast activity is stimulated by increased TGF-β1 secretion [84]. 

Although the data are limited, it seems that estrogen and progesterone hormones have a role in the occurrence of TMJ OA by direct action on bone cells [85,86]. Thus, at the onset of the disease, an increased level of estrogen has a protective effect by inhibiting the Wnt pathway and the activity of osteoclasts [85]. Moreover, the increased level of progesterone, by inhibiting NF-kB activity, has a beneficial effect on the subchondral bone, decreasing bone resorption [86]. 

Osteocytes, the most abundant bone cells, have an important role in osteoclastogenesis, being sensitive to joint mechanical loading and producing RANKL [87,88]. By secreting degradative enzymes such as CTS and MMPs, osteocytes react to different mechanical forces and resorb the bone matrix, leading to perilacunar/canalicular remodeling [89]. Studies have shown that a decrease in this bone remodeling determined by osteocytes can favor the onset of OA [90]. 

**Figure 1:**
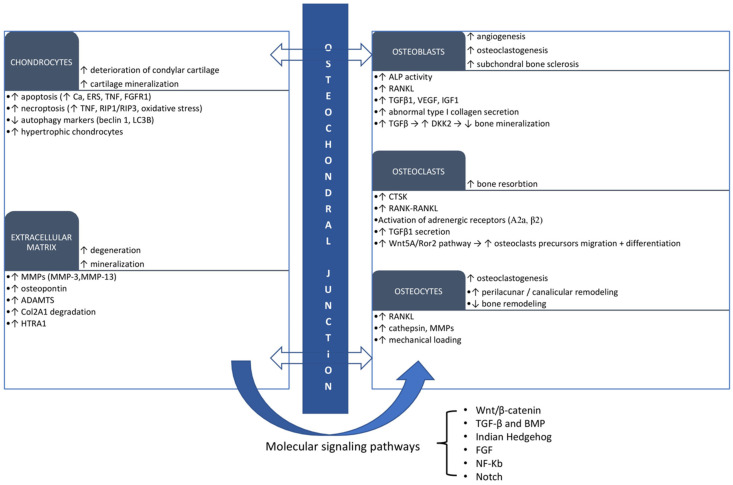
The role and the main metabolic changes occurring at the level of articular cartilage, osteochondral junction, and subchondral bone highlighted in TMJ OA. ALP—alkaline phosphatase; RANKL—receptor activator of nuclear factor kappa-B ligand; RANK—receptor activator of nuclear factor kappa-B; TGFβ1—transforming growth factor β1; VEGF—vascular endothelial factor; IGF1—insulin-like growth factor 1; DKK2—dickkopf-2; CTSK—cathepsin K; Wnt5A/Ror2—Wnt5A/receptor tyrosine kinase-like orphan receptor 2; MMPs—matrix metalloproteinases, ERS—endoplasmic reticulum stress; TNF—tumor necrosis factor; FGFR1—fibroblast growth factor receptor 1; RIP1/RIP3—receptor-interacting proteins 1 and 3; LC3B—light chain 3 beta; ADAMTS—a disintegrin and metalloproteinase with thrombospondin motifs; Col2A1—type II collagen A1; HTRA1—high-temperature requirement A1.

Figure 1 summarizes the role and the main metabolic changes occurring at the level of bone cells that have been highlighted in TMJ OA.

### 3.2. Inflammation, Subchondral Bone and Temporomandibular Osteoarthritis

Although OA is not a systemic inflammatory condition, data support the role of various pro-inflammatory cytokines in TMJ OA both at the level of articular cartilage and of subchondral bone [91,92]. In the synovial fluid of these patients, an inflammatory environment characterized by an increased secretion of many molecules such as IL (IL-1β, -2, -12, -17, -18), TNF (TNFα and TNFβ) and interferon (IFN)-γ was highlighted. Among all these, the main pro-inflammatory cytokine secreted in TMJ OA is IL-12 [93]. 

IL-1β and TNFα actively participate in inflammation by increasing the expression of chemokines, eicosanoids, and various proteins [94,95,96]. Moreover, through the stimulation achieved by IL-1β, TMJ synoviocytes increase their production of monocyte chemoattractant protein-1 (MCP-1) [97]. Recently published data support that MCP-1 can be considered the trigger for the appearance, progression, and persistence of inflammation even in the absence of IL-1β [98]. In addition, the secretion of these cytokines correlates with the suppression of the synthesis of the articular cartilage matrix [99]. 

TNFα, IL-1β, and IL-17 directly modulate osteoclast production, as well as bone resorption, by increasing RANKL secretion at the level of osteoblasts and fibroblasts from the synovial membrane [100]. The results of a study on mice support the role of TLR4 in the occurrence of TMJ OA. Thus, through the MyD88/NF-kB activation pathway, TLR4 favors the appearance of OA changes, in particular, the degradation of both cartilage and subchondral bone [101].

Inflammation also plays an important role in the development of joint pain, a frequent symptom in these patients. Pro-inflammatory cytokines such as TNFα or IL-1β can directly stimulate nociceptive receptors and cause sensory neuron hyperexcitability [102,103]. Other molecules such as growth factors, proteoglycans, or proteases seem to be involved in the development of arthritic pain, MMP-3 being considered a hallmark for TMJ pain [104]. Moreover, promising results on murine studies were published, pointing to the important role of macrophage/microglia activation in the development of pain in these patients [105].

Cytokines such as IL-1β and IL-6 can facilitate the transcription of VEGF in the nucleus, thus leading to an increased expression of VEGF [58,106]. IL-1β has the ability to directly activate NF-kB, while IL-6, through ERK1/2, activates ERRγ [26,58,106,107]. 

VEGF is the main molecule responsible for angiogenesis, a pathogenic mechanism involved in the development of TMJ OA and characterized by the formation of new blood vessels at the osteochondral junction [53,54,108,109]. By invading the articular cartilage, it determines both the formation of mineral deposits at the level of the ECM as well as the hypertrophy of chondrocytes. In addition, it participates in endochondral osteogenesis by incorporating new blood vessels into the osteophytes [110]. In addition to angiogenesis, an important role is played by neurogenesis, both processes favoring the appearance of OA and being involved in the appearance of joint pain [111]. Angiogenesis and neurogenesis influence each other through vascular and nerve growth factors, leading to the emergence of neurovascular interaction involved in the TMJ OA progression [112,113]. 

**Figure 2:**
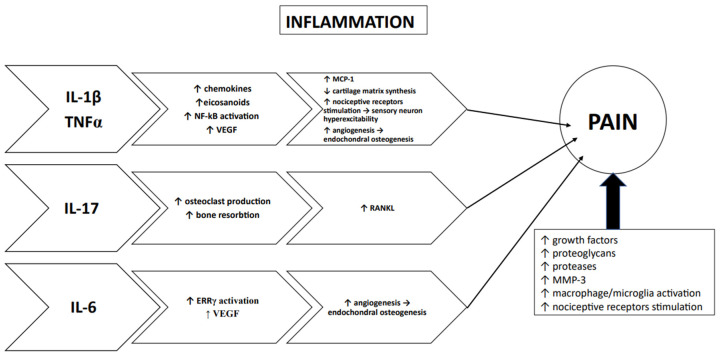
The role of inflammation in the changes in the subchondral bone and the development of arthritic pain. IL-1β—interleukin 1β; TNFα—tumor necrosis factor α; IL-17—interleukin 17; IL-6—interleukin 6; VEGF—vascular endothelial growth factor; MCP-1—monocyte chemoattractant protein-1; RANKL—receptor activator of nuclear factor kappa-B ligand; ERRγ—estrogen-related receptor γ; NF-kB—nuclear factor kappa-light-chain-enhancer of activated B cells.

Figure 2 shows the important role of inflammation in the changes at the level of the subchondral bone as well as its role in the occurrence of TMJ OA pain.

### 3.3. Mechanical Loading and Temporomandibular Osteoarthritis

The mechanical loading distributed on the surface of a joint is very important to maintain joint integrity and functionality, the bone microarchitecture being correlated with the direction and magnitude of the applied load [114]. Thus, the structure of the subchondral bone changes, highlighting changes such as: the increase in bone volume, the decrease in mineralization, the thickening of plates, and the decrease in the trabecular rod/plate ratio [115]. This is mainly due to the osteocytes that participate in the remodeling of the ECM through the increased secretion of degradative enzymes, as well as through the increase in their metabolic activity that leads to canalicular remodeling [116,117,118,119]. 

Although the TMJ is not a joint on which high mechanical load forces act, it seems that small alterations of the mechanical loading cause the appearance of degenerative changes [120]. The anatomical and positional changes in the fibrocartilaginous disc between the condyles and the joint fossa can be considered a cause of TMJ OA [121]. 

Another mechanism involved in the occurrence of TMJ OA is the decrease in the sensitivity of chondrocytes to mechanical loading, this being due to the knockdown of high mobility group protein B2 (HMGB2) [122]. Along with these, an abnormal subchondral bone remodeling characterized by a decrease in type I collagen production and increased bone resorption is involved in the development of TMJ OA [63]. Secondary to the increased resorptive activity, the data obtained from experimental models support the formation of new bone at the level of the condyles in the initial stages of degenerative changes [123]. 

## 4. Therapeutic Strategies Impacting Cartilage and/or Bone Changes in TMJ OA: Platelet-Rich Plasma, Hyaluronic Acid, and Stem Cell-Based Therapy

Presently, the most widely used treatment strategies in TMJ OA involve the diminishment of symptoms, with a focus on pain relief and improvement of mobility [124,125,126,127,128,129]. Nevertheless, recent studies support the regenerative or protective potential of certain therapies such as platelet-rich plasma (PRP), HA, and mesenchymal stem cells (MSCs) in OA [130,131,132]. The currently available data regarding the regenerative or protective potential of PRP, HA, and MSC-based therapy results from studies that used various treatment regimens and different assessment methods, which may partly explain the sometimes diverging findings.

### 4.1. Platelet-Rich Plasma (PRP)

In vitro research indicates that PRP (different formulations of autologous blood derivatives with a high concentration of platelets—cells involved in tissue healing) may bolster the proliferation of chondrocytes and MSCs with the concomitant deposition of type II collagen, thus potentially leading to cartilage repair [133]. Intraarticular PRP was proven effective in OA involving various joints, with studies showing that the treatment may significantly improve symptoms over various follow-up periods [134,135,136,137].

Diverse types of management options including PRP have shown promising results in in vitro and animal studies. Constructs of PRP embedded in different types of scaffolds and placed at the site of a cartilage defect where it exhibited positive effects on chondrocyte disposition, metabolic activity, glycosaminoglycan deposition, and collagen type II expression, as well as anti-apoptotic properties. Moreover, PRP was also used as a carrier for mesenchymal stem cells [138,139]. The molecular mechanisms through which PRP may contribute to tissue regeneration include its bioactivity. In this respect, growth factors such as TGFβ, IGF, VEGF, PDGF (platelet-derived growth factor), and bFGF (basic fibroblast growth factor) are known to be abundant in PRP and may encourage the proliferation of chondrogenic cells and the secretion of cartilaginous matrix components [140,141]. The development of PRP-based formulations for OA is based on several hypotheses including the potential anti-inflammatory and anti-catabolic properties of chemokines, and the anabolic effects of the growth factors found in PRP [142]. PRP inhibits serine/threonine kinase 1 (AKT1), phosphatidylinositol-4,5-bisphosphate 3-kinase (PI3K), and NF-kB (PIK/AKT signaling) and hinders the release of pro-inflammatory cytokines such as TNFα and IL6 [143,144].

Recently published studies involving patients with TMJ OA suggest that PRP may induce condylar bone repair according to imaging findings (MRI—magnetic resonance imaging and/or CBCT—cone beam computed tomography) [145,146]. In animal models of TMJ OA, PRP therapy was associated with the improvement of the joint structure histological aspect (Table 1) [147,148,149]. Local injections of PRP have also shown beneficial effects on pain relief and improved maximal mouth opening in patients as well as animal models of TMJ OA [150,151,152,153].

Animal studies based on the histological assessment of the TMJ cartilage and bone showed that intraarticular PRP leads to a restoration of the fibrocartilage and an improved microarchitecture of the subchondral bone [147,148,149]. AbuBakr et al. also observed a decrease in MMP and pro-inflammatory cytokine values following treatment, with a concomitant upregulation of type II collagen gene expression in experimental animals [149]. Arafat and Kamel found that combined treatment with PRP + HA may be associated with superior results compared to PRP alone [147].

The imaging findings in patients with TMJ OA indicated that PRP could improve the aspect of the condylar bone [145,146]. Nevertheless, these studies did not use the same evaluation methods in all patients (either CBCT or MRI).

### 4.2. Hyaluronic Acid (HA)

Intraarticular HA injections have largely been used to reduce OA-related symptoms in larger joints such as the knees [154,155,156]. Apart from its viscoelastic behavior, HA has shown the ability to support cell growth and the chondrogenic differentiation of stem cells, as well as to provide binding sites for growth factors, favoring tissue healing. Certain HA formulations were associated with decreased pro-inflammatory cytokine levels and MMPs [157,158]. An important topic in cartilage tissue engineering has been designing HA hydrogel formulations to enhance regeneration. A sulforaphane-loaded hyaluronic acid hydrogel demonstrated protective effects against cartilage degradation by reducing the depletion of proteoglycans, increasing collagen type II, while also modulating NF-κB [159]. Nevertheless, a great number of recent studies focus largely on the biomaterials used as scaffolds or carriers, rather than on the properties of HA itself [160,161,162].

A significant improvement in pain levels and functionality was also seen in patients with TMJ OA following HA treatment [15,163,164]. HA therapy has also shown some beneficial effects regarding the regeneration of joint structures in TMJ OA in both patients and animal models (Table 2). However, some studies did not support the restorative effects of HA in TMJ OA [165,166,167,168,169,170].

While some animal research suggested that HA could exhibit certain restorative or protective properties, in patients with TMJ OA, imaging findings (CBCT or MRI) indicated that HA alone does not improve the aspect of the cartilage or condylar bone [165,166,167]. However, the methodology and the treatment regimens varied greatly between studies.

Notably, the combination treatment of HA and PRP in a murine model of TMJ OA exhibited better results with respect to the regeneration of joint structures compared to HA alone [147]. Moreover, the addition of HA to MSCs may improve cartilage repair according to the results obtained by Köhnke et al. in a rabbit model of TMJ OA [168]. These findings suggest that HA could enhance the regenerative potential of other therapies (PRP, MSCs) rather than restore normal TMJ structures by itself.

### 4.3. Mesenchymal Stem Cells (MSCs)

MSCs may derive from a wide array of tissues such as the synovium, the umbilical cord, adipose tissue, dental pulp, and bone marrow. In recent years, the promising findings with respect to the important regenerative potential of MSC-based therapies in OA have been gathering interest [171,172,173].

Several mechanisms through which transplanted MSCs could restore the normal aspect of the arthritic joint have been proposed. In vitro studies indicated that, under controlled conditions, MSCs may differentiate into cartilage, bone, ligament, and tendon structures. Moreover, the exosomes (extracellular vesicles) released by these cells may carry various bioactive molecules. It has been shown that MSCs may exhibit immunomodulatory and anti-inflammatory effects and may promote angiogenesis [174,175,176]. MSCs could hinder the degradation of the cartilage extracellular matrix. Additionally, under the influence of MSCs, the macrophage-like synoviocytes may switch to the M2 phenotype, rather than the predominantly pro-inflammatory M1 by secreting prostaglandin-E2 and indoleamine 2,3-dioxygenase [177].

In TMJ OA, their use in patients and experimental animals was tied to numerous favorable effects including cartilage restoration and delayed degradation, chondrogenic and osteogenic differentiation, as well as the improvement in subchondral bone volume and structure. Moreover, MSCs have exhibited anti-inflammatory and trophic effects in TMJ OA in addition to potential pain reduction and improved functionality [178,179,180,181,182]. Research conducted in the last years describes histological changes and imaging findings suggesting joint structure regeneration following treatment (Table 3). However, studies differ in terms of the types of MSCs used, the treatment regimens and follow-up periods, as well as assessment methods [179,180,181,182,183,184,185,186,187,188].

Notable histological signs of cartilage regeneration were seen in animal models of TMJ OA (induced chemically, surgically, or mechanically or by a combination of chemical factors and mechanical stress). Moreover, some studies indicated that MSCs-based therapy could be superior to other treatment strategies for TMJ OA such as intra-articular injections of PRP and HA for the restoration of joint structures [149,168].

In patients with TMJ OA, imaging findings (CBCT, MRI) showed regenerative joint changes related to MSCs-based therapy [183]. In patients treated with MSCs, Carboni et al. described a better MRI aspect of the TMJ structures compared to controls receiving saline injections [184]. Based on CBCT evidence, Khairy et al. found that adipose-derived MSCs had better effects with respect to joint remodeling compared to HA injections [166].

However, in the study conducted by De Riu et al., there was no significant evidence of joint reparation following treatment with BMNC (bone marrow nucleated cell concentrate) according to the MRI scoring system [167].

## 5. Conclusions

Several potential mechanisms of TMJ OA have been hypothesized in the literature. The majority of these purported processes are predicated on the assumption that joint overloading, excessive pressures, and trauma all have a role in disturbing the structural integrity of the TMJ structure. Understanding the chain of biological processes that culminate in TMJ cartilage and bone degeneration is the first step in developing a complete model of the pathophysiology of TMJ OA.

Certain treatment strategies are linked to positive outcomes regarding the regeneration of joint structures over time in TMJ OA. While studies used diverse treatment regimens and evaluation methods, more prominent findings were described in MSCs-based treatment and PRP compared to HA. According to recent findings, the latter could enhance the restorative potential of other therapies (PRP, MSCs) when used in combination, rather than repair TMJ structures by itself.

TMJ OA is a complex disease in which degenerative changes in the cartilage and bone develop through intricate mechanisms. The regenerative potential of such therapies as PRP, MSCs, and HA regarding the cartilage and subchondral bone (alone or in various combinations) in TMJ OA remains a matter of further research, with studies sometimes obtaining discrepant results.

## Figures and Tables

**Table 1:** Recent evidence from human and animal studies regarding the effects of PRP therapy on cartilage and/or bone changes in TMJ OA.

Study	Study Description	Study Group(s)	Assessment	Main Findings: Changes Impacting the TMJ Cartilage and/or Bone
Liu et al., 2022 [145]	Comparison between PRP + physical therapy and PRP injections alone in patients with TMJ OA	Group 1: PRP injection Group 2: PRP injection + individualized comprehensive physical therapy	CBCT and MRI at baseline followed by CBCT or MRI post-therapy	Condylar bone repair was seen in both groups (78.6% for PRP alone, and 81.5% for the combined treatment). There was no significant difference between the two groups with respect to imaging findings.
Arafat and Kamel, 2021 [147]	Analysis of the effects of PRP and/or HA injections in a rat model of TMJ OA	Group 1: Controls Group 2A: HA alone (2 injections at 7 and 21 days) Group 2B: PRP alone (2 injections at 7 and 21 days) Group 2C: HA + PRP (2 injections at 7 and 21 days)	Histological assessment	The group treated with PRP alone exhibited a restoration of the fibrocartilage covering the entire surface of the condyle (yet with a decreased thickness) and a normal architecture of the subchondral bone. The combined treatment group showed better results, with a restored fibrocartilage layer and a normal aspect of the subchondral bone.
Coskun et al., 2019 [148]	Evaluation of PRP injections on cartilage and subchondral bone in a rodent model of TMJ OA (MIA-induced TMJ OA in rabbits)	Group 1: Single intraarticular injection of PRP Group 2: Three weekly injections of PRP Controls: Isotonic saline injections	Histological assessment	An improvement was observed in the treated animals. However, there were no statistically significant differences between the treatment groups and the controls.
Li et al., 2021 [146]	Retrospective comparison between PRP and chitosan injections in patients with TMJ OA	Group 1: PRP injections Group 2: Chitosan injections	CBCT or MRI	PRP treatment was associated with better results with respect to condylar bone repair, yet there was no statistically significant difference between the treatment groups.
AbuBakr et al., 2022 [149]	Comparative analysis of bone marrow MSC-derived microvesicles (BM-MSCs-MVs) and PRP in a murine model of TMJ OA (MIA-induced)	Group 1: No therapeutic intervention Group 2: BM-MSCs-MVs Group 3: PRP	Histological assessment	The TMJ condyle structure was improved after treatment with PRP compared to the MIA-induced TMJ OA animals. Additionally, there was a diminishment in inflammatory cytokine values, as well as MMP3 and MMP13, and an increased type II collagen gene expression in both treatment groups.

CBCT: cone beam computed tomography; MIA: monoiodoacetate; MMP: matrix metalloproteinase; MRI: magnetic resonance imaging.

**Table 2:** Recent evidence from human and animal studies regarding the effects of HA injections on cartilage and/or bone changes in TMJ OA.

Study	Study Description	Study Group(s)	Assessment	Main Findings: Changes Impacting the TMJ Cartilage and/or Bone
Arafat and Kamel, 2021 [147]	Analysis of the effects of HA and/or PRP injections in a rat model of TMJ OA	Group 1: Controls Group 2A: HA alone (2 injections at 7 and 21 days) Group 2B: PRP alone (2 injections at 7 and 21 days) Group 2C: HA + PRP (2 injections at 7 and 21 days)	Histological assessment	The group treated with HA alone demonstrated an incomplete restoration of the fibrocartilage layer and the regeneration of the subchondral bone. The combined treatment group showed the best results, with a fully formed fibrocartilage and a normal aspect of the subchondral bone.
Iturriaga et al., 2021 [169]	Comparison between high and low molecular weight HA injections (HMW-HA versus LMW-HA) in a rabbit model of TMJ OA (MIA-induced)	Group 1: No therapeutic intervention (healthy TMJs, TMJ OA) Group 2: 30-day follow-up (TMJ OA, LMW-HA, HMW-HA) Group 3: 135-day follow-up (TMJ OA, LMW-HA, HMW-HA)	Histological assessment	TMJ OA worsened without treatment. Both treatment groups exhibited cartilage and disc repair at 30 days, with better results in rabbit TMJs treated with LMW-HA. Nonetheless, a regression of tissue repair was seen in both treatment groups at 135 days.
Tolba et al., 2020 [170]	Evaluation of HMW-HA treatment in a rodent model of TMJ OA (CFA-induced)	Group 1: Controls (not injected) Group 2: Disease (CFA-induced arthritis) Group 3: Treated (CFA-induced arthritis + 3 weekly injections of HMW-HA)	Histological assessment	The HMW-HA treatment group demonstrated restoration of the normal histological aspect of the TMJ (with respect to the condyle, the subchondral bone, and the articular disc).
Cen et al., 2022 [165]	Retrospective comparison between HA injections and oral glucosamine + diclofenac in TMJ OA patients	Group 1: Four biweekly injections of HA Group 2: Oral glucosamine + diclofenac	CBCT	There was no statistically significant effect on bone changes in any of the treatment groups.
De Riu et al., 2019 [167]	A randomized clinical trial of HA compared to BMNC autograft treatment in patients exhibiting degenerative changes in the TMJ	Group 1: Arthrocentesis + BMNC Group 2: Arthrocentesis + HA	MRI	There was no significant imaging evidence of regeneration in the HA group according to MRI scores.
Khairy et al., 2019 [166]	Comparative analysis of HA injections versus chondrogenic differentiated adipose-derived MSCs in patients with TMJ OA	Group 1: Arthrocentesis + HA injection Group 2: Arthrocentesis + chondrogenic differentiated adipose-derived MSCs	CBCT	The HA-treated group showed no imaging evidence of restoration of the joint structures.
Köhnke et al., 2021 [168]	Analysis of treatment with HA and MSCs (separately and in combination HA + MSCs) in a rabbit model of TMJ OA (mechanical + collagenase-induced)	Group 1: AB serum Group 2: HA injection Group 3: MSCs Group 4: MSCs + HA	Histological assessment Back-scattered electron imaging (BEI)	Cartilage thickness was better in the HA treated group compared to the AB serum animals, with results approaching statistical significance. The HA + MSCs intervention showed the best results regarding cartilage thickness. According to BEI findings, there were no significant differences between the study groups with respect to subchondral mineralization.

CBCT: cone beam computed tomography; CFA: complete Freund’s adjuvant; MIA: monoiodoacetate; MRI: magnetic resonance imaging.

**Table 3:** Recent evidence from human and animal studies regarding the effects of MSCs on cartilage and/or bone changes in TMJ OA.

Study	Study Description	Study Group(s)	Assessment	Main Findings: Changes Impacting the TMJ Cartilage and/or Bone
Gomez et al., 2021 [185]	Analysis of cartilage regeneration after MSCs + PRP treatment in mice with surgically induced TMJ condyle cartilage damage (CCD, extended until the subchondral bone) in a murine model	Group 1: Human MSCs embedded into preclotted PRP Group 2: Untreated animals with TMJ condylar cartilage damage Group 3: Sham group	Histological analysis	At 6 weeks, the macroscopic aspect of the treated lesions displayed cartilage-like tissue occupying the damaged sections. The authors noted that a collagen and GAG matrix with chondrocytes filled the treated areas.
Khairy et al., 2019 [166]	Analysis of HA injections versus chondrogenic differentiated adipose-derived MSCs in patients with TMJ OA	Group 1: Arthrocentesis + HA injection Group 2: Arthrocentesis + chondrogenic differentiated adipose-derived MSCs	CBCT	Compared to the HA group, the patients treated with chondrogenic differentiated adipose-derived MSCs demonstrated CT evidence of remodeling.
Kim et al., 2019 [186]	Evaluation of the effects of different concentrations of human umbilical cord-derived MSCs in a rabbit model of TMJ OA (MIA-induced)	Group 1: Controls Group 2: Untreated TMJ OA Group 3: Dexamethasone Group 4–6: Low, Median and High concentrations of MSCs (chondrogenic differentiation) at 4 weeks post-induction of TMJ OA	Histological assessment Micro-CT	The authors described the regenerative effects (including potential chondrogenesis) in all MSC concentrations. The median dose of MSCs exhibited the best results regarding cartilage protection and restoration. Moreover, MSC treatment demonstrated potent anti-inflammatory effects.
Köhnke et al., 2021 [168]	Analysis of treatment with MSCs and HA (separately and in combination) in a rabbit model of TMJ OA (mechanical + collagenase-induced)	Group 1: AB serum Group 2: HA injection Group 3: MSCs Group 4: MSCs + HA	Histological assessment Back-scattered electron imaging (BEI)	Cartilage thickness was significantly better in the MSCs only treated group compared to the AB serum animals and the combination MSCs + HA group was significantly, superior to both AB serum and the HA only group. However, there were no significant differences between the study groups with respect to subchondral mineralization according to BEI.
Zhang et al., 2019 [187]	Examination of human embryonic stem cell-derived MSC exosomes in a mouse model of TMJ OA (MIA-induced)	Group 1: Phosphate-buffered saline Group 2: MSC exosomes Group 3: Sham group	Micro-CT Histological assessment	MSC exosome treatment demonstrated reparative and regenerative effects in mice with TMJ OA according to the histological analysis, with more prominent changes being described at 8 weeks. The imaging findings indicated that MSC exosome therapy had a beneficial effect on bone volume and structure.
Ogasawara et al., 2020 [188]	Analysis of intravenous stem cells from human exfoliated deciduous teeth (SHED-CM) effect in a mouse model of TMJ OA (mechanical stress-induced—forced maximal mouth opening 3 h/day for 5 or 10 days)	Group 1: Mechanical stress for 5 days (pre-treatment group) Group 2: Mechanical stress for 10 days + intravenous SHED-CM (days 6–10) Group 3: Mechanical stress for 10 days + intravenous DMEM (days 6–10)	Micro-CT (high-resolution) Histological assessment	SHED-CM therapy appeared to stimulate tissue repair and regeneration. The high-resolution micro-CT findings in the TMJ condyles of experimental animals showed that the SHED-CM group had a smoother surface of the cartilage and a reduced subchondral bone resorption.
De Riu et al., 2019 [167]	A randomized clinical trial of BMNC autograft treatment compared to HA injection in patients with degenerative changes in the TMJ	Group 1: Arthrocentesis + BMNC Group 2: Arthrocentesis + HA	MRI	There was no imaging evidence of regeneration related to BMNC treatment according to the MRI scoring system.
AbuBakr et al., 2022 [149]	Comparative analysis of bone marrow MSC-derived microvesicles (BM-MSCs-MVs) and PRP in a murine model of TMJ OA (MIA-induced)	Group 1: No intervention Group 2: BM-MSCs-MVs Group 3: PRP	Histological assessment	The condylar structure was improved after treatment with BM-MSCs-MVs. Moreover, there was a decrease in inflammatory cytokine levels, as well as MMP3 and MMP13, and an increased type II collagen gene expression. BM-MSCs-MVs were found to be superior to PRP in some respects.
Carboni et al., 2019 [184]	Evaluation of abdominal adipose tissue-derived MSCs in patients with internal derangement of the TMJ	Group 1: Adipose-derived MSCs Group 2: Saline injection	MRI	The MRI findings showed restoration of the joint structures in the treatment group.

CBCT: cone beam computed tomography; GAG: glycosaminoglycan; DMEM: Dulbecco’s Modified Eagle Medium; MIA: monoiodoacetate; MMP: matrix metalloproteinase; Micro-CT: micro-computed tomography; MRI: magnetic resonance imaging.

## Data Availability

Not applicable.
